# On Tic Douloureux, and the Best Mode of Treating It

**Published:** 1825-10

**Authors:** Powell Charles Blackett

**Affiliations:** Surgeon R.N.; Surgeon Extraordinary to H. R. H. the Duke of Clarence.


### ART. VIII.—*On Tic Douloureux, and the best Mode of Treating it.* By POWELL CHARLES BLACKETT, Surgeon R.N.; Surgeon Extraordinary to H. R. H. the Duke of Clarence.

### PART I.

THE tic douloureux is a violent, feverless, spasmodic affection of the nerves, particularly of the face, and occasionally of the extremities; but rarely of any other part, unless it be exposed to the influence of the external air. The attack is sometimes slight and gradual, and generally without any warning; although some patients feel a numbness in the parts, or a kind of sensation peculiar to the disease, which they cannot express, and which occasionally even produces palpitation and difficulty of breathing. The paroxysm, after a short time, becomes extremely excruciating; the pain, or spasm, rapidly vibrating, and shooting from one extremity to the other of the nervous branches or communications. These attacks continue sometimes only for a moment, or a few minutes. When a remission takes place, it is frequently of no longer duration; or, should it be prolonged for days, months, or even years, every renewed attack of the complaint will be found to bring with it an increase of pain. Dr. S. Fothergill says, "there is no determinate period; we always find the utmost irregularity in the same patient."

The paroxysms of pain vary according to the injury sustained by the part, and by the continuance of the disease. In slight cases the pain is supportable, and the patient has periods of rest from his sufferings; but in the more aggravated cases, brought on by serious causes, the intensity of torment is so great as frequently to produce appalling cries, distortion of the countenance; protruded tongue; corrugated forehead; the cheeks drawn backwards towards the ears, either on one side or the other, or both at the same time; the mouth occasionally fixed to one side, exhibiting the most shocking appearance, causing difficulty of speech and of swallowing; the eyes red and watery; attended occasionally with copious ptyalism, and continued convulsive and writhing agitations of the adjacent muscles, with more or less of pervigilium.

It is very rare that this complaint attacks both sides of the face at the same time, but it is sometimes known to affect each alternately. It is also found to rage more during the day than the night; which can only be accounted for by the better protection which bed and a warm room afford the patient from atmospheric influence; whilst conversation and mastication have always been observed to accelerate a return of the paroxysm.

Dr. S. Fothergill observes, that "the pain does not always confine itself to the seat of the disease, but darts with the rapidity of lightning to the neighbouring parts, shooting in different directions, like radii from a centre. It rarely gives warning of its approach; and often the first sign of an attack, is the patient starting up in a state little short of phrensy. In this condition, some patients beat the part with violence, or forcibly rub it with some rough substance till excoriation takes place; and in some instances they have succeeded in diminishing the intensity of the pain."

Moreover, as the symptoms which have been mentioned, and which affect patients in different degrees, according to their more robust, more vascular, or more irritable constitutions, so the modes of attack, which are perceived in this malady, vary also.

In some patients it will continue for several years, without much injury to the whole constitution, and without inducing a determination directly fatal; whilst in others it will emaciate the body and destroy the mind, from the want of rest, nourishment, &c. and disfigure the affected side of the countenance in a very singular manner.

Most authors, it is probable, agree in respect to the most frequent seat of this spasmodic nervous affection: it will, therefore, be found that, when the second branch of the fifth pair of nerves is affected, the pain will be either over the os malæ, just below the orbit, or in the ala nasi, the upper lip, the teeth, or the gums; but, when the ophthalmic, or first branch of the fifth pair, is the seat of the disease, it will then be found to affect the lachrymal gland, causing an effusion of scalding tears. The ophthalmic branch, which divides itself into two ramifications—the frontal and the nasal, is rarely the seat of this affection. The first is distributed to the muscles surrounding the eye, and the muscles and integuments of the forehead; the other (or the nasal) passes obliquely through the orbit, throwing one or two twigs to the fasciculi of the ciliary nerves, and thence is continued between the superior oblique and adductor muscles, through the internal orbital foramen; and, after again entering the cranium, it passes once more out through the cribriform plate of the ethmoid bone, and is extended to the superior spongy bones and frontal sinuses. When the third branch of the fifth pair is diseased, or the inferior maxillary nerve, the lower jaw, the under lip, the tongue, and the throat, are affected.

The portio dura of the seventh pair of nerves is as frequently diseased as any of the others, owing, no doubt, to the communication which its branches form with most parts of the face. The distinguishing mark, therefore, of the affection of the portio dura is, that the ear, the mastoid process, and the angle of the lower jaw, are principally attacked. Hence, from the connexion that exists between the fifth and the seventh pair of nerves, the disease will not continue long without extending its ravages, and affecting at last the whole of the nerves which are distributed throughout the face and head. This conjunction of ramifications, by which the minute branches communicate with the great sympathetic, explains why the face is so often affected in organic diseases.

### PART II.

*The predisposing causes.*—This disease as frequently attacks men as women. There are many authors who disagree on this subject; but, if we examine the cases on record, we shall find them nearly equal in point of number. It is seldom found—I may say never—in any person before the age of puberty, and in very few until the age of forty or fifty. This malady appears only to commence at that stage of life when the strength and functions of the constitution begin to be impaired, and the frame feels incipient decay. Yet, at the same time, there must be some peculiar predisposing cause, which is at present clouded, and will, I fear, remain in obscurity. Could this secret be discovered, the attacks of this most distressing malady might probably be averted.

*Exciting causes.*—The exciting causes may be divided into external and internal.

The external are, vicissitudes of temperature, exposure to cold or heat in damp situations, tempestuous and stormy weather; the sudden application of cold water when heated and fatigued; a gust of cold air blowing suddenly on the face; external injuries, wounds, blows, contusions, cicatrices; extraction of teeth, the irritation of artificial teeth; the application of caustic to sores; extraneous substances; repelled erysipelas, suppressed perspirations, and cold during the use of mercury. To these may be added the slighter cause or causes which bring on returns of the paroxysm,—such as talking, eating, drinking, shaving, blowing the nose, touching or rubbing, even in the most gentle degree, the parts which had once been affected. Any one of these is sufficient to bring back, in an instant, the paroxysm, and to cause the most distracting and excruciating torture.

"When," says Dr. S. Fothergill, "the pain has continued, with frequent accessions, for a length of time, a most distressing scene is sometimes witnessed. The patient, whose health at the time is generally good, after desisting from eating and drinking till the keenness of his appetite and the intensity of his thirst are too irresistibly urgent to be longer unrelieved, attacks whatever food is placed before him, with maniac fury and hurried precipitancy; his countenance suffused with crimson, and convulsed and tortured with pain. This horrid conflict does not last long: he soon throws down his knife and fork with desperate violence, obliged to solicit a cessation of pain by a state of inaction."

The internal exciting causes are, cancer; cessation of the catameniæ in the female; affections of the liver, kidneys, bladder, and urethra; diseased prostate gland, and obstruction, or the presence of irritating substances in the stomach or alimentary canal; with the various passions of anger, grief, &c. and great proclivity to venery.

PART III.

*Diagnosis.*—For the better elucidation of this part, it may be proper to add some remarks on the nervous system. The inherent power or contraction of the nerves is effected by certain applications, or excitants, made either to the nerves or muscles connected with them; and, in either case, the effects of such excitants so exactly assimilate, as to leave it difficult to decide whether the disease is first caused by nervous or muscular irritability. The nervous system has been pointed out to us as the substratum, or principal stamina in our body: in fact, our whole frame is principally nervous; there is no part of it but is either supplied with the sentient or the moving extremities of the nerves. I mean by the *sentient extremities,* those parts of certain nerves in which the medullary substance is divested of the enveloping membranes from the pia mater, and so framed as to be affected by certain excitants only; and, by the *moving extremities*, I mean those parts of certain nerves capable of certain and peculiar contractility or vibration; the contracting or vibrating power being caused alone by some very peculiar excitant. Therefore, when this disease has taken firm hold of the system, let it be from either one cause or the other, there cannot be any doubt or difficulty in distinguishing it. Its character, its symptoms, and its nature, are so strongly marked, that, when completely established, there is no practitioner, I hope, that can mistake them. But there are other cases which, at first sight, may be mistaken for this disease; and on these I wish to offer some remarks, as in all cases, whatever may be the complaint, it is necessary that the practitioner should decide quickly.

It is distinguished from cancer, as in cancer there must be tumor in the part; but, if there be cancerous acrimony in the constitution, the tic douloureux may still terminate in cancer of the lip, nose, or cheek, if the parts be irritated, or from the cancerous acrimony alone, though it had not before manifested itself.

It differs from scrofula, on the same grounds as cancer; but yet scrofula may be one cause of the disease, (especially if the bones are affected,) by the pressure or irritation of the nerves that pass through them.

From arthritic acrimony; as the pain disappears as soon as the gouty paroxysm commences.

From exanthematous acrimony; as, by the use of proper remedies, the pain soon ceases.

From catarrh, or rheumatism, or odontalgia; as these diseases strike more deep, and continue; whilst, in the tic douloureux, the paroxysm is short, the succession rapid, and the interval free from any kind of pain whatever.

From syphilitic acrimony; as in this the pain is unremitting, severer at night, and seated deep, apparently in the bones, and not the nervous or muscular parts.

From hemicrania, and various painful affections of the face and head which have their intermissions: these often occur in damp and stormy weather, and are in general caused by constipated bowels.

To conclude, all abscess, tumor, and antrum cases have symptoms so decidedly different, that no practitioner, acquainted with anatomy, can possibly mistake them for the tic douloureux; of which, a darting pain, vibrating through the various branches of the nervous ramifications, constitutes the marked character.

*Prognosis.*—The prognosis will ever be more unfavourable when the disease arises from violent injuries of the nerves, or organic affections, (which of themselves are in general incurable,) than when proceeding from cold, heat, or climate; when it comes on suddenly, and quickly advances to the most violent stage, than when slow in its progress; when the spasmodic affections quickly succeed each other, and are excited by very slight causes, than when there is a considerable interval.

Although there may be an objection to the introduction of cases, yet, as I consider all information respecting this disease to be important, I will add a few which have come under my own treatment.

CASE I.—The Rev. W. F—, aged about forty, of a thin habit, had been seized, in less than an hour after the act of preaching, with a very violent pain affecting the second branch of the fifth pair and portio dura of the seventh. At first his complaint was treated for rheumatism; but the symptoms showing themselves afterwards more distinctly, it was pronounced to be the tic douloureux. Various remedies were applied, both internally and externally, but without any effect. I then had recourse to the arsenical solution, which, being continued for six or seven weeks, relieved the violent symptoms, and would no doubt have effected a perfect cure, had not its disagreement with the bowels required its cessation. In its place, full doses of sulphate of zinc were administered, which in a few weeks more completely conquered his disorder.

The patient had no organic disease, and there can be no doubt but that his complaint was brought on by too sudden exposure to the external air, when fatigued and heated by the exertions of preaching and the warmth of a close and crowded chapel.

CASE II.—Mr. J. B—, a youth of about sixteen years, (and one of many, I am sorry to relate, who was addicted to a certain solitary vice,) applied to me in 1810, complaining of violent darting and vibrating pains about the forehead, but principally in the situation of the first branch of the ophthalmic, or first branch of the fifth pair; so that, when pressure was applied to the part affected, the paroxysm was increased. He had also a slight affection under the left eye; but the pressure on that nerve did not produce the same violence of pain as on the former. I had for some months previously begged this young man, who was much debilitated in mind and body, to put himself under my care; but he as often refused. I questioned him particularly respecting his lamentable habit, and used all the arguments in my power to dissuade him from his vicious course; in which I at last succeeded. I then gave him sulphate of zinc and opium, keeping his bowels free at the same time; and in the course of five or six weeks I effected a perfect cure.

CASE III.—Mrs. M. C—, aged about forty-five years, a widow, about the autumn of 1815 applied to me, complaining of violent pain of face affecting the left side, which she ascribed to rheumatism; and her symptoms, which she detailed at full length, seemed to warrant her opinion. But, on further examination of the parts, and finding in particular that, when I pressed on the infra-orbital branch of the fifth pair, it increased the pain, and made it spread over the forehead to the back part of the head of the same side, I decided it to be the tic douloureux. As the paroxysms were very short, but frequent, I at first gave her bark and opium; afterwards I administered sulphate of zinc and copper, arsenic, conium, hyoscyamus, belladonna, and aconitum; but nothing seemed to answer the purpose. I proposed dividing the nerve, and the day was fixed for the operation; when, on the morning before it was to take place, she showed me a part of a tape-worm she had voided, about seven inches long. It then forcibly occurred to my mind, that the tic douloureux was caused by these irritating animals in the alimentary canal. I gave her two drachms of the rectified spirit of turpentine, with ten drops of tincture of opium, three times a-day, in a solution of gum acacia. After the second dose I gave her an aperient, which operated and brought away two more pieces,—one about nine inches, and the other four and a half long. I continued this remedy three times a-day, and an aperient an hour after the second dose. The pain, after the fifth dose, became evidently less, but still at times very violent. She continued her medicine; and, after the eighth dose, she voided three complete but small tænia,—one eighteen and a half inches, another eleven inches, and the last six and a quarter inches long.

After the last discharge of the worms until her death, she continued free from the symptoms of the tic douloureux.

Case IV.—S. M—, aged nineteen years, complained of pain of the middle finger of the left hand, about the end of the spring of 1816. The pain was first produced by pinching the finger, and chiefly situated between the first and second phalanges. It was a decided nervous affection, and it agreed in all its symptoms with tic douloureux. The finger was amputated, after all the remedies usual in these cases were tried. About twelve months after she applied to me, and said the next finger (the ring-finger) was affected with the same disease. The idea of another operation was revolting. I examined her minutely as to her habits of life, and found her to be of that unfortunate class of females who obtain their livelihood by prostitution. She told me that a young surgeon advised her, whenever she had any sore, &c. to destroy it by lunar caustic. From this information I saw my way, and put her immediately under a course of mercury, not to cause ptyalism, but to keep the mouth tender. No sooner was her mouth affected, than the pains gradually lessened, and at last totally ceased. I kept her mouth in this state about eight or nine weeks, giving her the infusion of sarsaparilla at the same time. I have not seen her since her cure.

Case V.—Mrs. A. S—, aged sixty-three, for several years had been afflicted with severe and acute attacks of rheumatism on both sides of the face, especially the left: it commenced after the change of life, which was nearly thirteen years before. I saw her the first time in October, 1816; she then complained of a pain on the left side of the face, more darting than usual. I examined the part, and found the portio dura very tender to the touch, so as to excite the most piercing cries, attended with the most distracting writhings, &c.

About four days after, she was attacked with erysipelatous inflammation, which lasted about a week; at which time the other side began to be affected, and continued alternating. She also mentioned that, after these attacks, which came on monthly, she had always a disagreeable discharge per vaginam. I ordered her aperients, and tonics with opium, which relieved her for some months; but in about four months it returned, with more violence in all its symptoms. I then thought it advisable to examine per vaginam; when I found the os uteri enlarged so much that I could introduce my finger with ease, and indurated and so extremely irritable that I was obliged to discontinue the examination. Her face, her whole frame, became convulsed. I immediately gave her a full dose of laudanum, which in the course of a few hours relieved the irritation, but not the tic douloureux in the face. Concluding that this disease was produced by a scirrhus of the uterus, I thought it advisable to follow the plan of Mr. Richard Carmichael, of Dublin, by giving her five grains of the carbonate of iron every four hours, and an opiate at bed-time; taking care at the same time to keep her bowels open by gentle aperients. She continued this plan for a month, when the paroxysms became less severe and frequent. The discharge had nearly ceased; her appetite was better, and her nights so composed that I thought it advisable to discontinue the opiate. After three weeks pursuing this treatment, I found her strength much improved. There was no discharge; and the paroxysms were seldom felt, except when she exposed herself to cold winds. I ordered her not to wash her face, but to use almond or olive oil every night and morning, and to wipe it off dry. The only benefit and principle of the application of the oil, was that it formed a kind of mask to exclude the influence of the external air.

Independent of these cases, I have had the opportunity of seeing the post-mortem examinations of three or four patients.

The first was a negro, aged forty-five. This man's complaint was brought on by a wound made by the thorn of the prickly pear, which injured the portio dura. He died in the most aggravated torments, about nine weeks after the wound had been perfectly healed; and the paroxysms of the tic douloureux did not commence until three weeks before his death. The nerves were accurately examined, but they had not the least diseased appearance, nor did their coats in the least show any signs of inflammation. The muscles certainly were more rigid than common. The brain was perfectly healthy, as well as all the viscera.

The second case was a woman, aged about sixty, who died in great dejection of mind and emaciation of body. The nerves and brain, as well as viscera, were perfectly healthy, excepting the womb, which was in the last stage of cancerous ulceration.

The third was a coachman, aged forty-six. The appearance of nerves, &c. were, as usual, healthy; but he had stricture of the urethra, and a far advanced diseased prostate gland.

The fourth case was similar to the first. It was produced by a wound; and the appearances were exactly the same.

### *Treatment.*

*Indications.*—1st. To remove causes that are obvious.

2d. To allay the inordinate action of the nervous system, or excite a new and powerful action; and thereby to supersede the original and morbid one.

In the treatment of this disease, physicians and surgeons have in general attempted cures according to their different theories. The followers, therefore, of Fothergill, Rahn, Lentin, Pagol, &c. have their different opinions and modes of treatment; and of their inefficacy we have abundant and melancholy proofs.

The most powerful antispasmodics and tonics have had their day. Those which have been said to procure ease taken internally, are bark and its preparations, opium, belladonna, aconitum, stramonium, cicuta, hyoscyamus, colchicum, mineral acids, sulphate of zinc, argentum nitratum, sulphate of copper, arsenic, and the carbonate of iron. Those used externally are belladonna, blisters, stimulating embrocations or liniments, caustic, issues, fomentations, cupping, leeches, frictions with mercurial ointment, electricity, and magnetism. But we shall find the instances very rare in which these remedies have effected a cure, unless by chance they have been applied so as to relieve the primitive cause of the affection; which has made medical men, from distressing experience, deny their efficacy altogether.

By the first indication, the causes of this complaint, which are obvious, must be removed: therefore, when the disease is the consequence of a puncture or small wound, or lacerations, or burns, or when it is supposed to originate from the partial division of a nerve, a free dilatation of the wound should be made. If arising from local irritation, the nervous communication with the brain should be boldly, if practicable, cut off.

In performing this operation, I should recommend the nerve to be cut down upon, and to dissect out a portion: at the same time the surgeon should be quite sure that the nerve he is going to divide is the affected one, and really the principal seat of the disease. I do not approve of the cutting down immediately through the nerves, &c. to the bone, as some of the nervous fibres may slip under the knife, and bring on, after the nerve has recovered itself, a recurrence of the disease, and that in a more violent degree, in consequence of the additional irritation.

By the second indication, it will be found necessary to allay the inordinate action of the nervous system, or excite a new and powerful one, and thereby to supersede the original and morbid. This is to be done by antispasmodics and tonics, with various outward applications, and also by general and topical bleedings, which must be used only according to the causes and constitutions of the patients.

When this malady proceeds from wounds, opiates and general or topical blood-letting, with occasional aperients, will alone be found necessary at first; but afterwards recourse must be had to tonics.

If the disease proceeds from constipated bowels, or irritating substances in the stomach or alimentary canal, aperients with occasional opiates, and tonics, will in general complete the cure.

I

If it be caused by congestion towards the head, its remedies are then certainly obvious to the practitioner.

If by cancer, scrofula, and the various internal diseases already mentioned, those complaints must be first relieved or alleviated, or the paroxysms will not be removed. The remedies suitable to these diseases must be applied, with the addition (if the constitution of the patient will allow) of those medicines that will palliate the spasm.

If produced by external causes, such as climate, cold, heat, &c. the necessary remedies, according to the symptoms and constitutions of the patients, must be first used, and then antispasmodics and tonics to finish the cure.

Lastly, if it be brought on by the various passions, those passions must be diverted; the mind must be amused by various objects, and kept as much as possible from the irritating impressions, as it is well known the effect which the mind has over the body.

I look upon electricity and magnetism as of little or no benefit, excepting in cases where they amuse the mind and operate on the imagination of the patients.

*11, Park-street, Grosvenor-square; August.*

